# 5-Chloro-8-hydr­oxy-6-methyl-1,4-naphthoquinone

**DOI:** 10.1107/S1600536809010137

**Published:** 2009-03-25

**Authors:** Daniel Teoh-Chuan Tan, Hasnah Osman, Azlina Harun Kamaruddin, Samuel Robinson Jebas, Hoong-Kun Fun

**Affiliations:** a School of Chemical Sciences, Universiti Sains Malaysia, 11800 USM, Penang, Malaysia; b School of Chemical Engineering, Universiti Sains Malaysia, Seri Ampangan, 14300 Nibong Tebal, Penang, Malaysia; c X-ray Crystallography Unit, School of Physics, Universiti Sains Malaysia, 11800 USM, Penang, Malaysia

## Abstract

The mol­ecule of the title compound, C_11_H_7_ClO_3_, is planar, with a maximum deviation of 0.0383 (10) Å from the naphthoquinone plane. An intra­molecular O—H⋯O hydrogen bond generates an *S*(6) ring motif. The crystal packing is stabilized by inter­molecular C—H⋯O hydrogen bonds. Short intra­molecular Cl⋯O [2.8234 (8) Å] and O⋯O [2.5530 (11) Å], and inter­molecular Cl⋯Cl [3.2777 (3) Å] contacts further stabilize the crystal structure.

## Related literature

For the biological activity of the related compound 7-methyl­juglone, see: Mahapatra *et al.* (2007 ▶); Van der Kooy & Meyer (2006 ▶). For the synthesis of 7-methyl­juglone from the title compound, see: Musgrave & Skoyles (2001 ▶); Mahapatra *et al.* (2007 ▶). For bond-length data, see: Allen *et al.* (1987 ▶). For graph-set analysis of hydrogen bonding, see: Bernstein *et al.* (1995 ▶). For the stability of the temperature controller used for the data collection, see: Cosier & Glazer (1986 ▶).
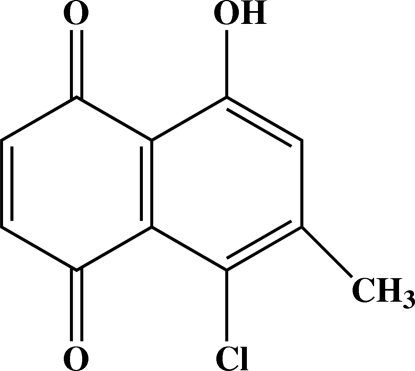


## Experimental

### 

#### Crystal data

C_11_H_7_ClO_3_
*M* *_r_* = 222.62
Monoclinic, 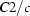

*a* = 10.7546 (1) Å
*b* = 10.3104 (1) Å
*c* = 16.8370 (2) Å
β = 100.285 (1)°
*V* = 1836.96 (3) Å^3^
*Z* = 8
Mo *K*α radiation
μ = 0.40 mm^−1^
*T* = 100 K
0.30 × 0.21 × 0.14 mm

#### Data collection

Bruker SMART APEXII CCD area-detector diffractometer
Absorption correction: multi-scan (*SADABS*; Bruker, 2005 ▶) *T*_min_ = 0.891, *T*_max_ = 0.945
17328 measured reflections
4015 independent reflections
3356 reflections with *I* > 2σ(*I*)
*R*_int_ = 0.031

#### Refinement

*R*[*F* ^2^ > 2σ(*F* ^2^)] = 0.038
*wR*(*F* ^2^) = 0.109
*S* = 1.07
4015 reflections
137 parameters
H-atom parameters constrained
Δρ_max_ = 0.61 e Å^−3^
Δρ_min_ = −0.35 e Å^−3^

### 

Data collection: *APEX2* (Bruker, 2005 ▶); cell refinement: *SAINT* (Bruker, 2005 ▶); data reduction: *SAINT*; program(s) used to solve structure: *SHELXTL* (Sheldrick, 2008 ▶); program(s) used to refine structure: *SHELXTL*; molecular graphics: *SHELXTL* software used to prepare material for publication: *SHELXTL* and *PLATON* (Spek, 2009 ▶).

## Supplementary Material

Crystal structure: contains datablocks global, I. DOI: 10.1107/S1600536809010137/sj2597sup1.cif

Structure factors: contains datablocks I. DOI: 10.1107/S1600536809010137/sj2597Isup2.hkl

Additional supplementary materials:  crystallographic information; 3D view; checkCIF report

## Figures and Tables

**Table 1:** Hydrogen-bond geometry (Å, °)

*D*—H⋯*A*	*D*—H	H⋯*A*	*D*⋯*A*	*D*—H⋯*A*
O3—H1*O*3⋯O2	0.86	1.73	2.5530 (11)	161
C2—H2*A*⋯O1^i^	0.93	2.51	3.4124 (12)	163
C3—H3*A*⋯O2^ii^	0.93	2.57	3.3000 (12)	136

Symmetry codes: (i) 
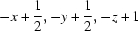
; (ii) 
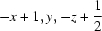
.

**Fig. 1.**
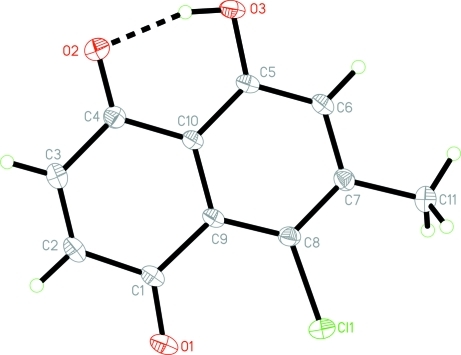
The molecular structure of the title compound, showing 50% probability displacement ellipsoids and the atom numbering scheme. The intramolecular H bond is drawn as a dashed line.

**Fig. 2.**
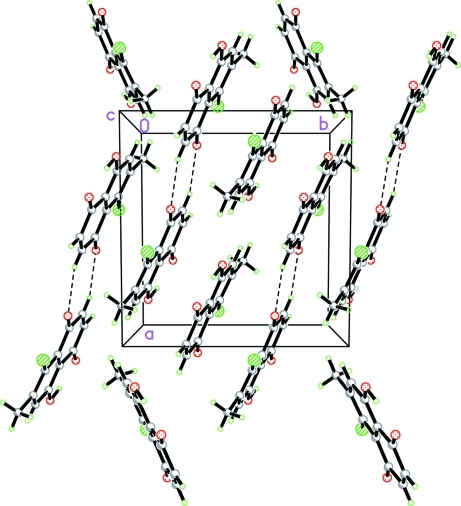
The crystal packing of the title compound, viewed along the c axis, showing dimer formation. Dashed lines indicate the hydrogen bonding.

## supplementary crystallographic information

### Comment

5-Hydroxy-7-methyl-1,4-naphthoquinone (7-methyljuglone) has recently been reported to exhibit activity against *mycobacterium tuberculosis* (Van der Kooy & Meyer, 2006; Mahapatra *et al.*, 2007). Naturally occurring 7-methyljuglone is synthesised from 8-chloro-5-hydroxy-7-methyl-1,4-naphthoquinone in high yield (Musgrave & Skoyles, 2001; Mahapatra *et al.*, 2007). This paper reports the molecular structure of 8-chloro-5-hydroxy-7-methyl-1,4-naphthoquinone; the precursor to synthetic 7-methyljuglone.

The asymmetric unit of (I) consists of one molecule of 8-Chloro-5-hydroxy-7-methyl-1,4-naphthoquinone. The napthoquinone ring is essentially planar with the maximum deviation from planarity being 0.0383 (10) Å for atom C8. The bond lengths in (I) have normal values (Allen *et al.*, 1987).

An intramolecular O–H···O hydrogen bond generates an S(6) ring motif (Bernstein *et al.*, 1995). The crystal packing is stabilized by intermolecular C–H···O hydrogen bonds (Table 2) (Fig 2). Short intramolecular Cl···O = 2.8234 (8) Å; O···O = 2.5530 (11)Å and intermolecular Cl···Cl^i^ = 3.2777 (3) Å [symmetry code: (i) 1 - *x*, *y*, 3/2 - *z*] contacts further stabilize the crystal packing.

### Experimental

The title compound was prepared from the Friedel-Crafts acylation of 4-chloro-3-methylphenol with maleic anhydride (Musgrave & Skoyles, 2001). Repeated Soxhlet extraction of the crude Friedel-Crafts product with n-hexane, and silica gel column chromatography purification [chloroform and n-hexane (1:9)] of the n-hexane extract afforded the title compound. Finally, slow evaporation of a n-hexane solution at 305 K gave single crystals of the title compound.

### Refinement

H atoms were positioned geometrically [C–H = 0.93 (aromatic) or 0.96Å (methyl)] and refined using a riding model, with *U*_iso_(H) = 1.2*U*_eq_(aromatic C) and 1.5*U*_eq_(methyl C). A rotating–group model was used for the methyl groups. The O bound hydrogen atom was located from the Fourier map and and refined isotropically with *U*_iso_(H) = 1.5*U*_eq_(C).

### Crystal data

**Table:**

C_11_H_7_ClO_3_	*F*(000) = 912
*M**_r_* = 222.62	*D*_x_ = 1.610 Mg m^−^^3^
Monoclinic, *C*2/*c*	Mo *K*α radiation, λ = 0.71073 Å
Hall symbol: -C 2yc	Cell parameters from 6307 reflections
*a* = 10.7546 (1) Å	θ = 2.8–30.1°
*b* = 10.3104 (1) Å	µ = 0.40 mm^−^^1^
*c* = 16.8370 (2) Å	*T* = 100 K
β = 100.285 (1)°	Block, red
*V* = 1836.96 (3) Å^3^	0.30 × 0.21 × 0.14 mm
*Z* = 8	

### Data collection

**Table:**

Bruker SMART APEXII CCD area-detector diffractometer	4015 independent reflections
Radiation source: fine-focus sealed tube	3356 reflections with *I* > 2σ(*I*)
graphite	*R*_int_ = 0.031
φ and ω scans	θ_max_ = 35.1°, θ_min_ = 2.8°
Absorption correction: multi-scan (*SADABS*; Bruker, 2005)	*h* = −12→17
*T*_min_ = 0.891, *T*_max_ = 0.945	*k* = −16→16
17328 measured reflections	*l* = −27→27

### Refinement

**Table:**

Refinement on *F*^2^	Primary atom site location: structure-invariant direct methods
Least-squares matrix: full	Secondary atom site location: difference Fourier map
*R*[*F*^2^ > 2σ(*F*^2^)] = 0.038	Hydrogen site location: inferred from neighbouring sites
*wR*(*F*^2^) = 0.109	H-atom parameters constrained
*S* = 1.07	*w* = 1/[σ^2^(*F*_o_^2^) + (0.0595*P*)^2^ + 0.6106*P*] where *P* = (*F*_o_^2^ + 2*F*_c_^2^)/3
4015 reflections	(Δ/σ)_max_ < 0.001
137 parameters	Δρ_max_ = 0.61 e Å^−^^3^
0 restraints	Δρ_min_ = −0.35 e Å^−^^3^

### Special details

**Table:**

Experimental. The crystal was placed in the cold stream of an Oxford Cyrosystems Cobra open-flow nitrogen cryostat (Cosier & Glazer, 1986) operating at 100.0 (1) K.
Geometry. All e.s.d.'s (except the e.s.d. in the dihedral angle between two l.s. planes) are estimated using the full covariance matrix. The cell e.s.d.'s are taken into account individually in the estimation of e.s.d.'s in distances, angles and torsion angles; correlations between e.s.d.'s in cell parameters are only used when they are defined by crystal symmetry. An approximate (isotropic) treatment of cell e.s.d.'s is used for estimating e.s.d.'s involving l.s. planes.
Refinement. Refinement of *F*^2^ against ALL reflections. The weighted *R*-factor *wR* and goodness of fit *S* are based on *F*^2^, conventional *R*-factors *R* are based on *F*, with *F* set to zero for negative *F*^2^. The threshold expression of *F*^2^ > σ(*F*^2^) is used only for calculating *R*-factors(gt) *etc*. and is not relevant to the choice of reflections for refinement. *R*-factors based on *F*^2^ are statistically about twice as large as those based on *F*, and *R*- factors based on ALL data will be even larger.

### Fractional atomic coordinates and isotropic or equivalent isotropic displacement parameters (Å^2^)

**Table:**

	*x*	*y*	*z*	*U*_iso_*/*U*_eq_	
Cl1	0.60008 (2)	0.08776 (3)	0.687132 (14)	0.02314 (8)	
O1	0.40351 (7)	0.18970 (8)	0.57005 (5)	0.02395 (16)	
O2	0.64859 (7)	0.18264 (8)	0.31989 (4)	0.02085 (15)	
O3	0.83772 (7)	0.06289 (8)	0.40048 (4)	0.02028 (14)	
H1O3	0.7847	0.1042	0.3653	0.030*	
C1	0.46492 (8)	0.18621 (9)	0.51561 (6)	0.01565 (16)	
C2	0.40771 (9)	0.23850 (9)	0.43571 (6)	0.01789 (17)	
H2A	0.3271	0.2743	0.4292	0.021*	
C3	0.46720 (9)	0.23648 (9)	0.37229 (6)	0.01852 (17)	
H3A	0.4275	0.2710	0.3232	0.022*	
C4	0.59410 (9)	0.18056 (9)	0.37912 (5)	0.01556 (16)	
C5	0.77541 (8)	0.06817 (8)	0.46273 (6)	0.01477 (15)	
C6	0.83488 (8)	0.01527 (9)	0.53621 (6)	0.01568 (16)	
H6A	0.9135	−0.0238	0.5394	0.019*	
C7	0.77959 (8)	0.01969 (9)	0.60416 (5)	0.01563 (15)	
C8	0.65972 (8)	0.07923 (9)	0.59849 (5)	0.01508 (15)	
C9	0.59512 (8)	0.12905 (8)	0.52543 (5)	0.01356 (15)	
C10	0.65466 (8)	0.12451 (8)	0.45647 (5)	0.01354 (15)	
C11	0.84754 (10)	−0.03753 (11)	0.68182 (6)	0.02193 (19)	
H11A	0.9265	−0.0738	0.6736	0.033*	
H11B	0.7964	−0.1044	0.6993	0.033*	
H11C	0.8632	0.0290	0.7222	0.033*	

### Atomic displacement parameters (Å^2^)

**Table:**

	*U*^11^	*U*^22^	*U*^33^	*U*^12^	*U*^13^	*U*^23^
Cl1	0.02101 (12)	0.03306 (14)	0.01720 (11)	0.00279 (9)	0.00841 (8)	0.00089 (8)
O1	0.0164 (3)	0.0311 (4)	0.0264 (4)	0.0055 (3)	0.0096 (3)	−0.0001 (3)
O2	0.0206 (3)	0.0258 (3)	0.0170 (3)	0.0004 (3)	0.0055 (3)	0.0009 (3)
O3	0.0172 (3)	0.0258 (3)	0.0200 (3)	0.0053 (3)	0.0093 (3)	0.0014 (3)
C1	0.0117 (3)	0.0146 (3)	0.0210 (4)	0.0004 (3)	0.0040 (3)	−0.0023 (3)
C2	0.0123 (4)	0.0162 (4)	0.0243 (4)	0.0018 (3)	0.0009 (3)	−0.0017 (3)
C3	0.0150 (4)	0.0188 (4)	0.0206 (4)	0.0015 (3)	0.0000 (3)	0.0006 (3)
C4	0.0146 (4)	0.0150 (3)	0.0170 (4)	−0.0012 (3)	0.0027 (3)	−0.0009 (3)
C5	0.0122 (3)	0.0152 (3)	0.0181 (4)	−0.0003 (3)	0.0058 (3)	−0.0017 (3)
C6	0.0115 (3)	0.0164 (4)	0.0193 (4)	0.0008 (3)	0.0034 (3)	−0.0012 (3)
C7	0.0126 (3)	0.0168 (4)	0.0171 (4)	−0.0006 (3)	0.0017 (3)	−0.0010 (3)
C8	0.0130 (3)	0.0174 (4)	0.0154 (4)	−0.0011 (3)	0.0043 (3)	−0.0013 (3)
C9	0.0103 (3)	0.0137 (3)	0.0172 (4)	−0.0002 (3)	0.0040 (3)	−0.0017 (3)
C10	0.0113 (3)	0.0141 (3)	0.0156 (3)	0.0001 (3)	0.0033 (3)	−0.0014 (3)
C11	0.0189 (4)	0.0279 (5)	0.0177 (4)	0.0024 (4)	−0.0001 (3)	0.0016 (3)

### Geometric parameters (Å, °)

**Table:**

Cl1—C8	1.7287 (9)	C5—C6	1.3980 (13)
O1—C1	1.2222 (12)	C5—C10	1.4092 (12)
O2—C4	1.2438 (11)	C6—C7	1.3812 (13)
O3—C5	1.3423 (11)	C6—H6A	0.9300
O3—H1O3	0.8581	C7—C8	1.4156 (13)
C1—C2	1.4777 (14)	C7—C11	1.5002 (13)
C1—C9	1.5008 (12)	C8—C9	1.3980 (13)
C2—C3	1.3393 (14)	C9—C10	1.4234 (12)
C2—H2A	0.9300	C11—H11A	0.9600
C3—C4	1.4670 (13)	C11—H11B	0.9600
C3—H3A	0.9300	C11—H11C	0.9600
C4—C10	1.4667 (13)		
			
C5—O3—H1O3	99.0	C6—C7—C8	118.69 (8)
O1—C1—C2	118.62 (8)	C6—C7—C11	119.62 (8)
O1—C1—C9	123.19 (9)	C8—C7—C11	121.69 (8)
C2—C1—C9	118.18 (8)	C9—C8—C7	121.48 (8)
C3—C2—C1	122.65 (8)	C9—C8—Cl1	122.56 (7)
C3—C2—H2A	118.7	C7—C8—Cl1	115.95 (7)
C1—C2—H2A	118.7	C8—C9—C10	118.71 (8)
C2—C3—C4	120.92 (9)	C8—C9—C1	123.27 (8)
C2—C3—H3A	119.5	C10—C9—C1	118.02 (8)
C4—C3—H3A	119.5	C5—C10—C9	119.69 (8)
O2—C4—C10	121.37 (8)	C5—C10—C4	119.08 (8)
O2—C4—C3	119.69 (8)	C9—C10—C4	121.21 (8)
C10—C4—C3	118.94 (8)	C7—C11—H11A	109.5
O3—C5—C6	117.51 (8)	C7—C11—H11B	109.5
O3—C5—C10	122.69 (8)	H11A—C11—H11B	109.5
C6—C5—C10	119.80 (8)	C7—C11—H11C	109.5
C7—C6—C5	121.57 (8)	H11A—C11—H11C	109.5
C7—C6—H6A	119.2	H11B—C11—H11C	109.5
C5—C6—H6A	119.2		
			
O1—C1—C2—C3	−178.75 (9)	O1—C1—C9—C8	−2.55 (14)
C9—C1—C2—C3	0.15 (13)	C2—C1—C9—C8	178.61 (8)
C1—C2—C3—C4	0.34 (14)	O1—C1—C9—C10	176.82 (9)
C2—C3—C4—O2	−178.28 (9)	C2—C1—C9—C10	−2.03 (12)
C2—C3—C4—C10	1.01 (14)	O3—C5—C10—C9	−178.76 (8)
O3—C5—C6—C7	178.08 (8)	C6—C5—C10—C9	1.06 (13)
C10—C5—C6—C7	−1.75 (13)	O3—C5—C10—C4	−0.34 (13)
C5—C6—C7—C8	0.12 (13)	C6—C5—C10—C4	179.48 (8)
C5—C6—C7—C11	−179.54 (9)	C8—C9—C10—C5	1.20 (13)
C6—C7—C8—C9	2.23 (13)	C1—C9—C10—C5	−178.19 (8)
C11—C7—C8—C9	−178.11 (9)	C8—C9—C10—C4	−177.18 (8)
C6—C7—C8—Cl1	−177.07 (7)	C1—C9—C10—C4	3.43 (12)
C11—C7—C8—Cl1	2.59 (12)	O2—C4—C10—C5	−2.09 (13)
C7—C8—C9—C10	−2.87 (13)	C3—C4—C10—C5	178.63 (8)
Cl1—C8—C9—C10	176.38 (7)	O2—C4—C10—C9	176.30 (8)
C7—C8—C9—C1	176.48 (8)	C3—C4—C10—C9	−2.98 (13)
Cl1—C8—C9—C1	−4.26 (12)		

### Hydrogen-bond geometry (Å, °)

**Table:**

*D*—H···*A*	*D*—H	H···*A*	*D*···*A*	*D*—H···*A*
O3—H1O3···O2	0.86	1.73	2.5530 (11)	161
C2—H2A···O1^i^	0.93	2.51	3.4124 (12)	163
C3—H3A···O2^ii^	0.93	2.57	3.3000 (12)	136

Symmetry codes: (i) −*x*+1/2, −*y*+1/2, −*z*+1; (ii) −*x*+1, *y*, −*z*+1/2.
